# The Supposed Curative Effect of Operations Per Se

**Published:** 1891-09

**Authors:** 


### Selections.

### "THE SUPPOSED CURATIVE EFFECT OF OPERATIONS PER SE."

Under this title Professor J. William White, of Philadelphia, contributes a paper to the *Annals of Surgery* for August, 1891, which not only from its subject, but from the great number of authorities quoted and from the peculiarly rich experience of the writer makes an article of unusual interest and importance to both surgeon and physician. The author's attention was first directed to this subject by reason of his experience with the operation of trephining for so-called traumatic epilepsy.

During the past five years, with Dr. D. Hayes Agnew, he has trephined in fifteen cases of supposed traumatic epilepsy. All but one recovered from the operation. The patient who perished was an imbecile and a confirmed drunkard as well as an epileptic. Death occurred from suppression of urine probably secondary to etherization.

In one case a bullet was found imbedded in the brain substance, in another an irregular portion of the internal table was dissected out from beneath the dura mater to which it was attached by cicatricial adhesions. In another there were projecting spicules of bone on the internal surface of the button removed and the adjacent portions of the skull. In two marked sclerosis and thickening of the cranium were observed about the field of operation. In remaining cases nothing abnormal was seen. Although this was the case they were without exception markedly improved by trephining ; in two instances even to the point of apparent cure, no return of symptoms having been observed for eighteen months, and for two years after the operation.

In the other seven the results were strikingly favorable, convulsions disappearing for weeks or months, although previously of more than daily occurrence.

* The author has, in so far as this is possible, classified the cases in which operation *per se* seemed to be the main factor in bringing about a cure. These cases are divided into three groups in accordance with the anatomical seat of the symptoms or of the supposed disease. This brings them under the following heads :

1. Operations for the relief of nervous phenomena, as epilepsy, insanity, paralysis, etc.

2. Operations for abdominal and pelvic disorders, as peritonitis, tumors, etc.

3. Miscellaneous Operations.

This classification is further carried out by grouping together, (a) Those cases in which nothing whatever was found explanatory of the symptoms. (b) Those in which some departure from normal conditions was observed, but was so slight as to be apparently inadequate to explain the symptoms. (c) Those cases in which an apparently grave and irremediable condition was disclosed by an exploratory operation, but notably improved or altogether disappeared after mere inspection or handling, no further surgical interference having been thought justifiable.

Under the heading of "Operations for the Relief of Nervous Phenomena," Dr. White had tabulated, including his own service, one hundred and fifty-four cases. Many of these are given in detail, and coming, as they do, from recognized authorities are of exceeding great interest.

In fifty-six cases of trephining for epilepsy nothing abnormal was found to account for the symptoms. Nineteen cases were reported in six months or less after operation ; eleven cases were reported from six to twelve months after operation; six cases were reported from one to two years after operation ; one was reported eight years after the operation.

Twenty-five of these cases were reported as cured, eighteen as improved ; in three cases it was mentioned that a relapse occurred later.

In thirty cases of ligation of blood vessels for epilepsy fourteen were reported as cured; fifteen as improved; one died seven days after operation. In the fatal case the right common carotid artery was tied. ' No fit occurred after the operation.

In ten cases of castration for epilepsy all were reported as cured. One case was reported four months after operation; four cases were reported more than two years after operation; in five the time when reported is not mentioned.

In nine cases of tracheotomy for epilepsy two were reported as cured; six as improved; one as much improved, though death in this case followed in two months after the operation.

In twenty-four cases of removal of the superior cervical ganglia of the sympathetic nerve six remained well at the end of three years; ten were improved; five remained unimproved; two died soon after the operation but not from its direct effects.

In six cases of incision of the scalp for epilepsy nothing was found to account for the symptoms. Three of these cases were reported as cured at the end of three months or less; one was reported as cured at the end of one year; two were reported as cured at the end of two years; two other cases almost similar were reported as cured.

Twelve cases of epilepsy are reported as cured by such operations as stretching of the sciatic nerve, excision of the musculo-cutaneous nerve, cauterization of the larnyx, circumcision, application of a seton to the back of the neck, tenotomy of the external recti muscles, burning of the scalp, puncture of the heart, etc.

Thirteen cases of spontaneous or accidental cures of epilepsy are also reported, at a time varying from two months to five years after the traumatism, which was a fall, a burn, a wound, an amputation for intercurrent injury or disease, etc.

Passing from the cerebral to the spinal region, Dr. White cites an illustrative case of his own. A man, aged fifty-five, was attacked on December 25, 1887, with severe pains in his arms and shoulders. A few days later there was weakness of the thighs spreading rapidly down the legs to the feet, and upward on the body to the nipple line. In eight days there was absolute paralysis of the parts involved, including both sphincters, while at the same time the paralyzed parts became the seat of profound anæsthesia. Girdle pains developed, bed sores made their appearance, percussion of the spine over the third and fourth vertebræ became painful. The reflexes were exaggerated, and light blows on the head in the direction of the spinal axis gave rise to frightful exacerbation of the girdle pains. In spite of every remedial measure these symptoms increased in severity for ten months. An exploratory operation was then undertaken. Dr. White removed the spines and laminæ of the first five dorsal vertebræ, opened the slightly thickened dura, separated some firm adhesions to the subjacent pia mater, explored the cord, and having failed to discover any serious pathological changes closed the wounds in the dura and soft parts.

The girdle pain had entirely disappeared by the following day, sensation began to return in the feet the day after, voluntary motion in the toes after the eighth day, and so one symptom after another disappeared, until the patient completely recovered and is now earning his living by manual labor.

In the list of abdominal and pelvic disorders apparently cured by operation *per se*, a number of extraordinary cases are cited. The experience of Tait, who has more than once drawn attention to the astonishing disappearance of tumors often of large size, after a mere exploratory incision, and the corroborative testimony of Von Mosetig are recited at length. Kœnig's analysis of one hundred and thirty-one cases of tubercular peritonitis treated by abdomonal incision is carefully discussed.

In response to letters of inquiry upon the subject, Dr. White received many communications from prominent operators, the great majority of them containing notes of cases not previously published.

Among the signers of these letters are to be found the names Goodell, Hirst, Battey, Roswell Park, Lusk, Cheever, Chas. T. Parkes, Cabot, Hunter McGuire, Nancrede, Weir, Stimson, and many others of equal note.

Under the heading of miscellaneons operations, the author has given several of very diverse character.

First are quoted osteo-malacia, cured, after weeks or months of confinement to bed, by either oöphorectomy or Cæsarian section.

Passing to another subject the question of graduated tenotomy of the eye muscles for the relief of severe nervous symptoms is carefully discussed. The author freely acknowledges the value of tenotomies both complete and graduated in the restoration of equilibrium in badly balanced ocular muscles, but he is none the less convinced that in numbers of instances of reported cures of chronic chorea, petit mal, and even delusional insanity, the effect of the operation *per se* is in large measure the potent cause of the supposed cure. This belief is founded not alone on theory, but upon the fact that in certain cases of reflex nervous trouble a cessation of the symptoms has followed the tenotomy, although this has not produced perfect equilibrium. Again, the relapses which may take place after a perfectly successful series of tenotomies would indicate that the nervous phenomena attributed to the insufficiency, for the relief of which the operations were made, were not correctly so attributed, and that the temporary relief must be ascribed to some cause other than the restoration of an imperfect balance of the external ocular muscles.

In seeking for a reasonable explanation of the phenomena observed in the above cases the author has formulated the conditions which are common to nearly all of them. These are:

1. Anæsthesia.
2. Psychical influence, or so-called mental impression.
3. Relief of tension.
4. Reflex action, or the correction of traumatism.

These influences were operative in the majority of cases, although not one of them except the last applies to the whole list.

With the idea that it was conceivable that a disease of the nerve centres, not reached by ordinary drugs might be affected by agents of such volatility and diffusibility as ether and chloroform, the author instituted a series of observations upon a number of epileptics in various stages of the disease. All other treatment was withdrawn, ether was given to the production of full anæsthesia at intervals of from forty-eight to seventy-two hours. The results were either entirely negative, or in consequence of the withdrawal of their bromides, the patients grew worse.

Since in the great majority of cases upon which Dr. White bases his paper, there were either undoubted symptoms such as are habitually associated with organic disease, or there was demonstrable and unmistakable evidence of such disease, it is necessary to believe, in considering the psychical influence of operation, that powerful impressions, acting upon the emotional or intellectual nature, may affect the organic processes of secretion, nutrition, etc., and may arrest pathological changes and bring about reparative or recuperative action. Cases are cited in which such influences are clearly set forth.

The author holds that the normal equilibrium which we witness between the cerebro-spinal and the sympathetic systems, as respects their influence upon the blood-vessel, is obviously more or less interfered with, when the brain transmits a more than wonted impulse, allowing the unrestrained action, or paralyzing the influences of the sympathetic vaso-motor nerve. In this relation the author narrates some remarkable cases of hypnotism, and quotes some striking examples of the effect of the central nervous system upon the body.

Belief is expressed that in many of the cases described there can be little doubt that relief of tension is an important factor in amelioration or cure. If it is assumed that preternatural tension exists in the cranial cavity, this would be relieved to an extent by trephining, and there would be but few exceptions to the rule that in each case something was done which lessened tension in the cavity or organ of the body. There are other cases, however, in which no such relief was obtained, and yet cure resulted from operation. A diminution of the tension would manifestly alter the blood supply to any important organ in the body, and with it the nutritive processes local and general. Beyond this nothing definite can be said except as it applies to cases of ascites in which as, in cases of hydrarthrosis, 1, tapping may prove permanently curative because the original source of irritation and hypersecretion has already disappeared.

Under the head of Reflex Action the author includes the "reaction of traumatism," as well as the effects of revulsion and counter-irritation.

Verneuil has long since shown that very slight traumatism sometimes excites in the entire economy a general perturbation, and sometimes, by selection of the weak point, a sudden aggravation of lesions that are only slight or have slumbered. This same excitement, usually prejudical, may occasionally be curative. In the case of spinal surgery, above detailed, Dr. White believes that the local shock of the operation was promptly followed by a corresponding reaction, in which the vitality of the tissues was raised sufficiently high to determine a return to the normal state. In this relation the reciprocal influence of one portion of the body on another is briefly discussed.

In considering abdominal tumors attention is called to the possibility of the spontaneous disappearance of such tumors, the relation of this disappearance to the operation being coincidental; cases are cited in point. As to the cure or amelioration of growths thought to be malignant by merely exploratory operation, a long search through the literature of the subject has met with but little success.

The cure of tuberculosis of the peritoneum as the result of exploratory incision is explained on the ground that the removal of ascitic fluid allows the peritoneal surfaces to fall together, and to acquire adhesions. The tubercles are then shut in between the coils of intestine, the omentum and the abdominal wall. They are thus surrounded by tissues in a high degree of activity, which can now throw around them the limiting zone of young cells, and eventually fibrous tissue, which if the tuberculous process is not too far advanced may effectually resist it, and may cause it to retrograde, the process being analogous to that which we see imperfectly going on around a cancerous growth.

As a result of a study of the subject the author believes the following conclusions are warranted :

1. There are large numbers of cases of different grades of severity and varying character which seem to be benefited by operation alone, some of them by almost any operation.

2. These cases include chiefly epilepsy, certain abdominal tumors, and peritoneal effusions and tubercle, though the improvement in the latter is, perhaps, to be explained on general principles.

3. Of the possible factors which, by reason of their constancy must be considered, anæsthesia seems least likely to have been effective. The other three, viz., psychical influence relief of tension, and reflex action, may enter in varying degrees into the therapeutics of these cases, and taken together serve to render the occurrence of occasional cures less mysterious.

4. The theory of accident or coincidence scarcely explains the facts satisfactorily.

---

THE ÆTIOLOGY OF DIPHTHERIA.—In the *Johns Hopkins Bulletin*, Dr. W. H. Welch gives the latest results of the researches on this point, and adds from his own studies still other observations. From all this he concludes it is fully proved that the specific primary cause of diphtheria is the Klebs-Loffler bacillus. This organism he calls the bacillus diphtheriæ. This bacillus is present in every case of primary diphtheria, in such number and situation as to explain the local manifestations of the disease. It can be isolated in pure culture readily, and a disease, identical in all respects with human diphtheria, can be produced experimentally by the inoculation of pure cultures.

Thus we are in possession of positive means for making a diagnosis of diphtheria. The method of doing this is not difficult and can readily be applied, though it may be questioned whether many practitioners are likely to make use of these means.

We are taught that there are pseudo-membranous anginas which must be separated from ætiologically pure diphtheria, and that diphtheria may exist in extremely mild forms even without visible pseudo-membranous deposits.

The endless controversy as to whether diphtheria is primarily a local or general disease is settled. It is primarily local, the grave constitutional symptoms are the result of intoxication with poisonous products, formed by the local action of the bacilli.

We can study the varied effects produced upon the animal body by the specific toxic products of the diphtheritic germ.

We can separate the alterations belonging to the disease itself from the many complications of the disease. Intelligent measures of prophylaxis can be based upon a definite knowledge of the characters of the specific germ and its behavior in the body. Rational indications for treatment can be established, and have been formulated.—*American Lancet.*

### MEDICAL ITEMS.

It is said that eleven physicians have found a specific for tuberculosis. The latest (by Max Schüller) is injections of iodoform, with the internal use of guaiacol.

The late Mr. W. A. Moore, of Atlanta, bequeathed $7,500 to the Grady Hospital, on condition that the institution be maintained by the city government.

The third annual meeting of the Tri-State Medical Association will convene in Turner Hall, Chattanooga, Tenn., Tuesday, October 27th, 1891, and continue in session three days. Indications are that it will be one of the largest medical meetings ever held in the South. Representative physicians from all sections will be present.

All who desire to read papers should send title to the Secretary of the Association before September 1st. In due time a circular will be issued, giving a complete list of all papers and names of exhibitors who apply for space before October 1st.

W. L. GAHAGAN,
Sec'y of Executive Committee,
*P. O. Box* 542. *Chattanooga, Tenn.*

